# A scoping review of Q-methodology in healthcare research

**DOI:** 10.1186/s12874-021-01309-7

**Published:** 2021-06-21

**Authors:** Kate Churruca, Kristiana Ludlow, Wendy Wu, Kate Gibbons, Hoa Mi Nguyen, Louise A. Ellis, Jeffrey Braithwaite

**Affiliations:** 1 Centre for Healthcare Resilience and Implementation Science, Australian Institute of Health Innovation, Macquarie University, Level 6, 75 Talavera Rd, Sydney, NSW 2109 Australia; 2 School of Psychology, University of Queensland, Brisbane, QLD 4072 Australia; 3 Australian Institute of Health Innovation, Macquarie University, Sydney, NSW 2109 Australia

**Keywords:** Q-methodology, Mixed methods, Healthcare, Qualitative methods, Factor analysis, Subjectivity, Patient perspectives

## Abstract

**Background:**

Q-methodology is an approach to studying complex issues of human ‘subjectivity’. Although this approach was developed in the early twentieth century, the value of Q-methodology in healthcare was not recognised until relatively recently. The aim of this review was to scope the empirical healthcare literature to examine the extent to which Q-methodology has been utilised in healthcare over time, including how it has been used and for what purposes.

**Methods:**

A search of three electronic databases (Scopus, EBSCO-CINAHL Complete, Medline) was conducted. No date restriction was applied. A title and abstract review, followed by a full-text review, was conducted by a team of five reviewers. Included articles were English-language, peer-reviewed journal articles that used Q-methodology (both Q-sorting and inverted factor analysis) in healthcare settings. The following data items were extracted into a purpose-designed Excel spreadsheet: study details (e.g., setting, country, year), reasons for using Q-methodology, healthcare topic area, participants (type and number), materials (e.g., ranking anchors and Q-set), methods (e.g., development of the Q-set, analysis), study results, and study implications. Data synthesis was descriptive in nature and involved frequency counting, open coding and the organisation by data items.

**Results:**

Of the 2,302 articles identified by the search, 289 studies were included in this review. We found evidence of increased use of Q-methodology in healthcare, particularly over the last 5 years. However, this research remains diffuse, spread across a large number of journals and topic areas. In a number of studies, we identified limitations in the reporting of methods, such as insufficient information on how authors derived their Q-set, what types of analyses they performed, and the amount of variance explained.

**Conclusions:**

Although Q-methodology is increasingly being adopted in healthcare research, it still appears to be relatively novel. This review highlight commonalities in how the method has been used, areas of application, and the potential value of the approach. To facilitate reporting of Q-methodological studies, we present a checklist of details that should be included for publication.

**Supplementary Information:**

The online version contains supplementary material available at 10.1186/s12874-021-01309-7.

## Background

Healthcare systems and organisations are recognised for their complexity. They involve a diverse number of stakeholders, such as healthcare professionals, patients, family members/informal caregivers, policymakers, insurance agencies, governments, professional bodies, community and charitable organisations, and the general public [1]. These individuals and groups interact and influence one another in the planning, delivery and receipt of care. Formalised examples of such interactions include multidisciplinary meetings [2], care coordination [3], and consumer involvement in service organisations [4]. However, stakeholder groups often have distinct perspectives and priorities on issues related to care delivery, even those that seem well-defined, such as the nature of illness, what constitutes appropriate treatment, or quality of care [5–8].

Understanding differing perspectives within healthcare is important because the ways in which individuals and groups makes sense of these issues affects their behaviours [9]. Research methods to apprehend perspectives have traditionally been qualitative (e.g., interviews and focus groups), though quantitative surveys have also been utilized to assess stakeholders’ attitudes. These approaches have their own well-described strengths and limitations [10–12]. Another approach that has gained recognition in healthcare research for identifying and comparing individuals’ and groups’ contrasting viewpoints is Q-methodology.

### Q-methodology: A mixed-method for studying perspectives

**Fig. 1:**
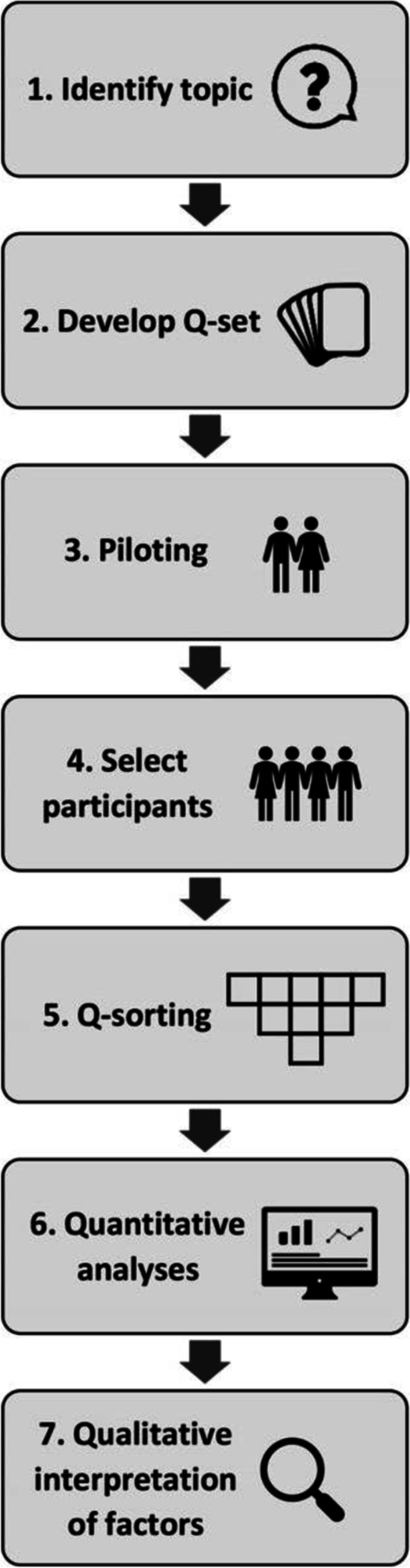
The stages of Q-methodology

Q-methodology combines qualitative and quantitative techniques to empirically study subjectivity [13]. It involves Q-sorting, where individuals articulate their own viewpoint by ranking a set of statements (the Q-set) about a particular issue based on some defined dimension, for example, level of agreement or perceived importance [14]. Each participant’s final ranking of statements is called their ‘Q-sort’ and these are analysed using inverted factor analysis techniques; ‘inverted’ because each participant (or their whole Q-sort) is treated as a variable, unlike factor analysis of surveys, where the items are variables. The analysis aims to identify patterns of similarity as well as differences in how participants have ranked, and therefore understood, the Q-set. Factors emerge as the commonalities across participants’ viewpoints and are qualitatively interpreted. The whole process of a Q-methodological study can be summarised in seven stages (see Fig. 1).

#### Stage 1: Identify the topic

Q-methodology identifies differences in what people think, as well as common viewpoints (shared meaning). Accordingly, suitable topics are those complex and socially contested subject matter where we expect to find both variation and commonality, for which we want to “hear ‘many voices’” [15].

#### Stage 2: Develop the Q-set

Developing the Q-set involves collection of discrete ideas, concepts, or usually statements about the topic, ideally until saturation is reached. In Q-studies, this is called sampling the concourse which refers to all the ideas and statements that can be sensibly expressed about that topic. Sampling might involve a literature review, preliminary data collection (e.g., interviews) or searching other publicly available resources [16]. Collected statements are then reduced and refined (e.g., grouping similar ideas together) to produce a manageable Q-set [17].

#### Stage 3: Piloting

Piloting provides researchers with insights into the ease with which instructions and statements are understood, the time taken to complete the Q-sort, and participants’ overall impression of Q-sorting. These may lead to modifications to the study materials or procedure [17].

#### Stage 4: Participant selection

Q-methodology uses purposive sampling; participants are selected because they are able to articulate a viewpoint on the topic of interest and because their perspective matters [14, 15]. In quantitative surveys, larger samples are required to ensure representativeness and generalisability [18, 19]. On the other hand, because Q-methodology uses an inverted factor analysis and does not aim to generalise to a population, studies typically have samples of around 40–60 participants [14, 15].

#### Stage 5: Data collection—Q-sorting

Data collection involves a card-sorting task called ‘Q-sorting’, where participants rank the statements in the Q-set. To begin, participants are provided with a clear question or prompt about the topic and then asked to respond by ordering the statements according to some level of judgement (e.g., “most strongly disagree” to “most strongly agree”). Q-sorting is typically conducted on a fixed quasi-normal distribution grid, which facilitates ranking and formalises the tendency for individuals to feel relatively strongly about fewer issues [14]. Some studies have incorporated think-aloud tasks [20] during Q-sorting [21–23], in order to capture participants’ thoughts about different statements and their decision-making processes in real-time. Following completion of their Q-sort, participants are usually asked post-sorting questions about the process, as well as about their background and experience of the topic in general; this may lead into the use of other empirical methods (e.g., interviews).

#### Stage 6: Analysis—Quantitative analyses to obtain factors

Factor analysis is a statistical method for classifying variables (here Q-sorts) into factors through the identification of sizable portions of shared meaning [14]. Factors are found by correlating each Q-sort with every other one to determine the extent to which they have a similar configuration of the Q-set. Q-sorts with a lot in common are typically subsumed under the same factor. Technical aspects of analysis in Q-methodology are similar to traditional factor analysis, with a number of different options for factor extraction (centroid, principal component) and rotation (varimax, by-hand) available depending on the research question and nature of the study.

#### Stage 7: Interpretation—Qualitative interpretation of factors

Qualitative factor interpretation is assisted by the construction of factor arrays—ideal Q-sorts computed for each factor based on a weighted averaging of the participants’ Q-sorts that loaded on the factor. The overall configuration of statements in a factor array is more important than the placement of a few particular items (e.g., most negative/positive). Interpretation involves developing narratives for each factor that incorporate as many statements from the factor array as possible and may draw on other data of participants who loaded on that factor.

### Q-methodology in healthcare research

Q-methodology was developed by psychologist William Stephenson in the early part of the twentieth century in the United Kingdom (UK) [24, 25]. Unlike surveys and conventional factor analysis, it had little take-up until much later, with the publication of a seminal text discussing its value to political science [26]. More recently, a number of authors have highlighted the value of Q-methodology for healthcare research. In 1997, Valenta and Wigge [27] introduced Q into health informatics, where it was used to measure physicians’ and medical students’ propensity to adapt to information technology in their workplace. Almost a decade later, Cross [28] argued for the value of studying attitudes in health-related fields using Q-methodology, noting how one’s subjectivity fundamentally impacts on one’s own behaviour. In 2008, Akhtar-Danesh, Baumann et al.[29] put forward a case for using Q in nursing research to study attitudes, feelings, values and life experiences, a theme then picked up by Simons [30] in 2013. More recently, Q-methodology has been advanced as an approach for studying priorities and priority-setting in healthcare [21, 22, 31–33]. Despite this, the extent to which Q-methodology has been utilised in healthcare, how, and for what purposes, remains unclear. Such information on current applications would not only provide insights into emerging conventions for its use in healthcare, but also demonstrate to what extent Q-methodology has value in this field.

A scoping review was selected as the most appropriate review approach because the focus was to examine how research is conducted within a defined field (Q-methodology in healthcare) and identify key characteristics of this body of research [34]. The aim of this scoping review was to explore how Q-methodology has been used in healthcare research. Specifically, this study answered the following research questions:

1. For what reasons is Q-methodology used in healthcare?
2. What healthcare topic areas are explored or studied using Q-methodology?
3. What types of participants are involved in these studies?
4. What materials are used in these studies?
5. How has Q-methodology been applied in healthcare studies? (i.e., details of the methods).
6. What are the results and potential implications of these Q-methodological studies?

## Methods

A protocol was developed to cover all stages of this scoping review and was agreed upon by KC and KL prior to conducting the search, but not published. The reporting of this review was guided by the Preferred Reporting Items for Systematic Review and Meta-analyses extension for Scoping Reviews (PRISMA-ScR) checklist [35]. The checklist for the PRISMA-ScR is provided in Additional file 1.

### Search and information sources

In January 2020, Scopus (Title-abstract-keywords), EBSCO-CINAHL Complete (title, abstract, subject headings) and Medline (Web of Science topic search) were searched using the terms outlined in Table 1. The full search for Medline is provided in Additional file 2. No date restrictions were applied to the search. Data records were downloaded into EndNote X8 and duplicates were removed.

**Table 1:** Search strategy

Keywords
1. q methodology OR q-methodology OR q method OR q-method OR q sort OR q-sort
AND
2. health care OR healthcare OR health-care OR medic^a^ OR nurs^a^ OR health services OR patient OR hospital^a^ OR clinic^a^ OR acute care OR primary health OR primary care OR general practice

^a^ indicates truncation

### Eligibility criteria and study selection

The inclusion criteria were: a) English-language, b) a peer-reviewed journal article; c) focuses on healthcare; d) constitutes a full Q-methodological study of (i) Q-sorting (e.g., card-sorting techniques) AND (ii) inverted factor analysis; f) full-text is available. ‘Healthcare’ included perspectives on health issues that impact care provision, recognised sites of healthcare delivery, or involvement of patients and healthcare professionals, including the education of healthcare professionals. Studies of health, i.e., individuals’ personal perspectives on wellbeing or their health conditions were excluded if not also related to healthcare delivery.

To establish inter-rater reliability, KC and KL completed a blinded title and abstract review of a random 5% sample of publications. A title and abstract review of remaining publications was conducted by KC. Included articles then underwent a full-text review by KC, KL, WW, KG and HMN. Regular discussions were had between team members to ensure consistency of article inclusion.

### Data collection processes and data items

Data items were extracted from included articles into a purpose-designed Microsoft Excel spreadsheet and included: publication details; country(ies) where data was collected; study aim; healthcare topic; reasons for using Q-methodology; methods for devising the Q-set; number of statements; ranking anchors; delivery method; analysis method, rotation and program used; other methods used; number of participants and participant group description; number and names of factors; variance explained; and study implications.

### Synthesis of results

The synthesis of results was descriptive in nature. Frequency counts were used to summarise the findings, which were then organised by data items. An iterative process of coding was used to develop a classification system for the broad healthcare topic areas and for study authors’ reasons for using Q. Throughout the results section of this review, examples are used from included studies to illustrate how Q-methodology has been used in healthcare research.

## Results

### Search

**Fig. 2:**
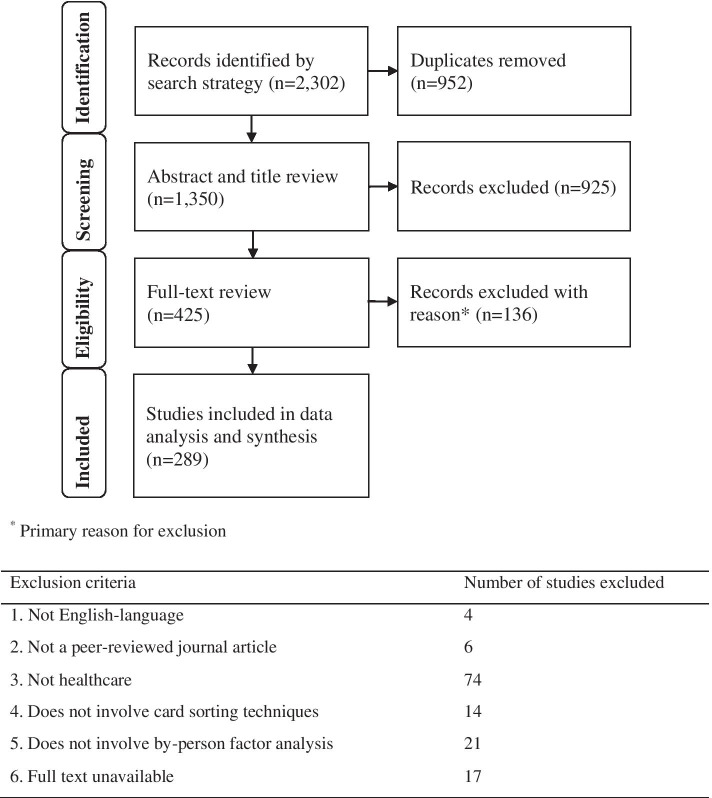
PRISMA diagram of search and review process [37]

A total of 2,302 publications were identified by the search strategy, with 1,350 publications included for screening and review after removal of duplicates. Cohen’s kappa for the 5% blinded double screening of abstracts was calculated as 0.82, which is considered a ‘strong’ level of agreement [36]. Figure 2 details the retention of studies after the abstract and title review and the full-text review. A total of 289 studies were included for data analysis and synthesis. Full details of all included studies are provided in Additional file 3.

### Characteristics of included articles

**Fig. 3:**
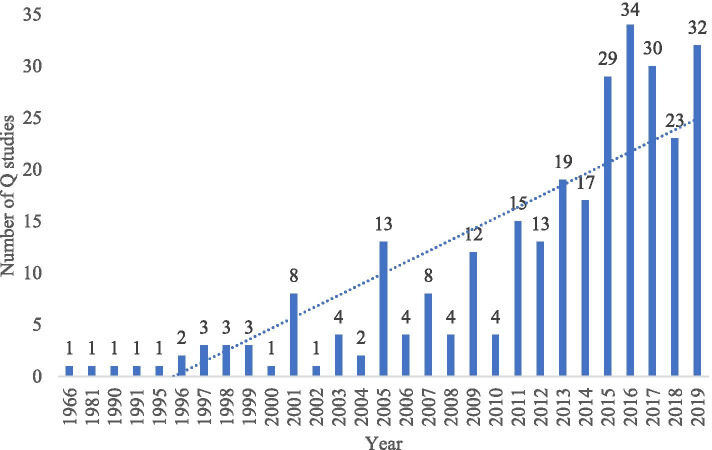
Included studies year of publication

Studies were published between 1966 and 2019 (Fig. 3), across 188 different journals. The journals publishing the most healthcare-related Q-methodology studies were: the Journal of Advanced Nursing (n = 13), Nurse Education Today (n = 9), Patient Preference and Adherence (n = 6), Psychology and Psychotherapy: Theory, Research and Practice (n = 6), the Indian Journal of Science and Technology (n = 5), Patient Education and Counseling (n = 5) and Nursing & Health Sciences (n = 5).

**Fig. 4:**
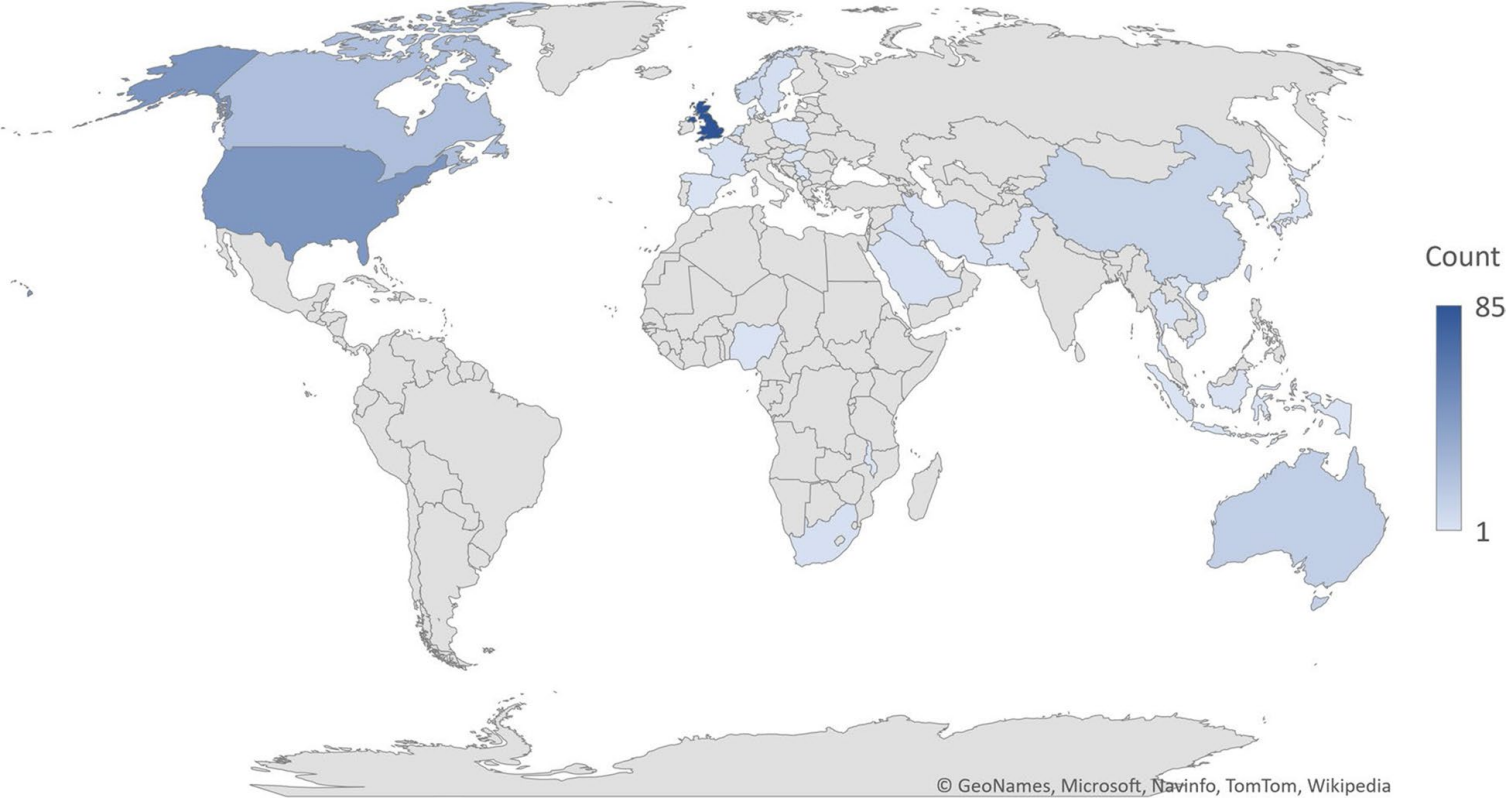
Geographic distribution of countries publishing Q-methodology studies. Microsoft product screen shot(s) reprinted with permission from Microsoft Corporation

The included studies had been conducted in 34 countries (Fig. 4), with four studies [32, 38–40] reporting on multiple countries. Nine studies did not state which country the research had been conducted in [41–49]. The majority of studies were conducted in the UK (n = 85), followed by The United States (US) (n = 46), South Korea (n = 40), Canada (n = 22), the Netherlands (n = 22) and Australia (n = 12).

### Reasons for using Q-methodology

A diverse range of reasons were provided for using Q-methodology. These justifications were grouped into five categories: exploring complex issues, taking a holistic approach, useability of results, rigour, and practicalities.

#### Exploring complex issues

Q-methodology was described as an ideal method for exploring complex or controversial topics [50] and topics that were likely to elicit strong opinions, differing perspectives, or underacknowledged views, something which is particularly pertinent to healthcare [51–54]. For example, Askay et al. [42] reasoned that standardised and relatively objective assessments, such as questionnaires, may not be appropriate to assess the physical, emotional and social reasons for distress in burn survivors because they are unlikely to be able to quantify the complex interactions among the reasons. Q-methodology was also used due to its less-confronting nature (i.e., participants are not asked direct questions but rather they respond to pre-established statements) [55]. Some studies justified that Q-methodology had been successfully used in previous studies of similar participant groups or topics; for example, Clarke and Holt’s disability-related research [56] and Grijpma et al.’s research with kidney transplant patients [57].

#### Taking a holistic approach

Regarding the study approach, Q-methodology was said to be a person-centred, holistic approach that provided a ‘wholeness’ of view [50, 58, 59]. This was in part due to the forced-choice nature of the method. Some studies reported using Q-methodology as it requires participants to sort cards in relation to each other [60]. This meant that participants were not able to select the same response for each item, which was reasoned to more accurately portray real-life situations in which decision-making is constrained and must consider multiple factors [61]. The forced-choice answering style used by Q-methodology makes it likely to identify opposing opinions [62]. White et al. pointed out that other methods, such as surveys, often average opposing opinions, which can then be incorrectly interpreted as neutrality [63, 64]. Flurey and Morris [65] noted that patients with rheumatoid arthritis likely had variable daily life experiences; more traditional methods for collecting experience, however, tended to produce “overall consensus on these experiences (creating) the potential problem of providing a ‘bland generalisation’”. ^(page 365)^.

#### Useability of results

In terms of study outcomes, one of the most common reasons for using Q-methodology was that it enables subjectivity to be empirically studied by identifying distinct attitudes, motives, perspectives, beliefs, feelings, priorities, perceptions, views or typologies [57, 66–68]. For example, Jedeloo et al. [66] used Q-methodology to explore the preferences of adolescents with chronic conditions regarding self-management of care and hospital care. The study findings could be used to guide nurses’ interactions with adolescents with chronic condition and tailor care to their preferences.

#### Rigour

Another common reason given by study authors for using Q-methodology was its rigour. Q-methodology was said to allow participants to create their own meaning while reducing the impact of prior assumptions and the potential biases of the researcher [69, 70]. Many studies referred to the integration of qualitative and quantitative techniques as one of Q-methodology’s key strengths, [42, 71–73] combining the richness of qualitative data with the rigour of statistical analysis [74]. Q-methodology was also described as avoiding problems associated with missing data because Q-sorts are *only* analysed as completed wholes [75].

#### Practicalities

The practicalities of Q-methodology were a further justification for its use. For some studies this was because it only requires a small number of participants, which is useful when studying a minority participant group, such as operating room personnel [76] or mothers on a psychiatric Mother and Baby Unit [55]. Whyte and Smith reasoned that the boardgame-like nature of the method was familiar to adolescents and therefore would be fun for participants [77]. Q-methodology was said to allow participants to express their views non-verbally, [78, 79] which enabled patients with communication difficulties, for example, head and neck cancer patients, to participate [80].

### Topics of Q-studies in healthcare

Topic areas covered a broad range of settings and healthcare conditions (Table 2). The most common topic areas were health professional education (n = 50, 17.3%), nursing practice (n = 39, 13.5%) and mental healthcare (n = 37, 12.8%).

**Table 2:** Topic areas for studies using Q-methodology in healthcare

Topic Area	Count	%
Health professional education	50	17.3
Nursing practice	39	13.5
Mental healthcare	37	12.8
Chronic disease	18	6.2
Gender, sexual and reproductive health	16	5.5
End of life care	15	5.2
Health and healthcare attitudes	11	3.8
Cancer	10	3.5
Healthcare technology	9	3.1
Health system planning, resource allocation and access	8	2.8
Organisational values, behaviours and roles	8	2.8
Allied health	8	2.8
Dentistry and orthodontics	8	2.8
Primary care	7	2.4
Dementia and aged care	7	2.4
Pharmacy	7	2.4
Caregiving	6	2.1
Other^a^	25	8.7
Total	289	100

^a^ ‘Other’ covers categories with ≤ 5 studies and includes acute and infectious conditions, disability, medicine, organ transplant, paediatric care, population health, rehabilitation, and research practice

### Participants

Q-methodological studies in healthcare included a diverse range of stakeholders (e.g., patients, doctors, nurses, informal caregivers, medical students), which could be broadly classified as those receiving or consuming care (consumers) or those delivering or organising formal care (providers). Thirty studies (10.4%) [27, 53, 59, 64, 81–106] involved both consumer and provider participants. Four of these studies [87, 92, 105, 107] did not clearly report the breakdown between the types of participants. One other study involved a mix of consumers and providers, with some participants belonging to both categories [94].

#### Consumers

Consumers were participants in 123 studies (65.1%) and included previous and current patients from a variety of settings (e.g., hospitals, out-patient clinics, orthodontics, antenatal clinics, psychiatric settings, primary care), informal caregivers/family, people with various medical conditions, and members of the general public. Of those studies clearly specifying the number of consumers, the number of participants (P-set) ranged from 5–299 (Mdn = 33).

#### Providers

Care providers were participants in 196 studies (67.8%). The term ‘provider’ refers to health professionals (e.g., psychologists, nurses, GPs, allied health professionals, specialists, community health workers, medical residents), hospital volunteers, people working in a healthcare-related field (e.g., IT specialists or university faculty members), and students studying healthcare-related fields (e.g., nursing, medicine, physiotherapy). The most common occupations of participants were nurses (n = 76, 38.6%), GPs/Physicians (n = 48, 24.4%), and students (n = 42, 21.3%). Nursing was the most common study area for studies involving students (30 of 42 studies, 71.4%). Over a third of studies involving providers (n = 70, 35.7%) had participant samples comprising a mix of professions. Of the studies clearly specifying the number of care provider participants, P-sets ranged from 4–710 (Mdn = 39).

## Materials

### Sampling the concourse and developing the Q-set

The methods used to sample the concourse were varied, with most studies utilising multiple sources of information. These included reviewing academic literature (n = 186, 64.4%), conducting interviews (n = 138, 47.8%), seeking input from ‘experts’ in the field (n = 59, 20.4%), running focus groups or group discussions (n = 45, 15.6%), reviewing grey literature such as media sources and websites (n = 44, 15.2%), using a Q-set derived from a previous study (n = 33, 11.4%), and repurposing items from surveys or questionnaires (n = 19, 6.6.%). Other less common methods included video-taped counselling sessions, observations, and conducting document/audit reviews. Twelve studies (4.2%) did not report on how inputs for the Q-set were identified, or the reporting was unclear.

Methods to then reduce collected statements to a usable Q-set included piloting, expert review, thematic analysis and use of theoretical frameworks. The numbers of statements in a final Q-set ranged from 16–275 (Mdn = 42). Nine studies [65, 95, 103, 108–113] (3.1%) used multiple Q-sets for different participant groups. Two studies (0.7%) did not report the number of statements used.

### Ranking scales and anchors

Numerical rankings used to sort the Q-set ranged from -2 to + 2 (or + 1 to + 5) to -7 to + 7. The most common numerical ranking was -4 to + 4 (n = 109, 37.7%), followed by -5 to + 5 (n = 90, 21.1%). Twelve studies (4.2%) did not include a negative value, for example, + 1 to + 9. Two studies [65, 113] (0.7%) involved the administration of two Q-sets with different ranking scales. In Flurey et al.’s study of rheumatoid arthritis, [65] participants were first asked to sort 39 statements regarding their daily experiences of living with rheumatoid arthritis from least agree (-5) to most agree (+ 5). They were then asked to sort a different Q-set comprising 23 statements regarding their help-seeking behaviours, from least agree (-4) to most agree (+ 4). Tang and colleagues [113] asked orthodontic patients to sort 30 statements about the reasons for wearing braces, ranked from -4 to + 4. They also asked patients’ parents to sort 35 cards about the reasons for their children’s orthodontic treatment, ranked from -5 to + 5. Twenty studies (6.9%) did not report the numerical ranking used.

Agreement was the most common anchoring term (n = 189, 65.4%), followed by importance (n = 26, 9.0%), priority (n = 4, 1.4%), characteristic (n = 3, 1.4%), significance (n = 3, 1.4%) and negative/positive (3%, 1.4%). Thirty-one additional anchors were used by either one or two studies. Twenty studies (6.9%) did not report on the anchor terminology used.

### Administration of the Q-sorting task

The majority of studies (n = 184, 63.7%) delivered the Q-sorting task face-to-face, with one study [114] allowing participants to take the Q-sorting task away and return their Q-sorts within 3 weeks. Eighteen studies (6.2%) administered the Q-sorting task via mail. Twenty-eight studies (9.7%) administered the Q-sorting task online. An additional ten studies (3.5%) used multiple delivery methods; five studies [81, 89, 102, 115, 116] (1.7%) administered hard copies of the Q-sorting task both face-to-face and via mail, and five other studies [32, 59, 84, 106, 117] (1.7%) used both online and face-to-face administration. Forty-five studies (15.6%) did not explicitly state how Q-sorting was administered and four (1.4%) were unclear.

Of the 33 studies offering online administration, FlashQ (n = 12, 36.7%) was the most commonly used analysis program. Other programs included Q Assessor (n = 5, 15.2%), author-developed platforms (n = 4, 12.1%), Q-SortWare (n = 2, 6.1%), POETQ (n = 2, 6.1%), emailed PQS files (n = 1, 3.0%), Q-SORTOUCH (n = 1, 3.0%), QSort (n = 1, 3.0%), QSortOnline (n = 1, 3.0%), HTMLQ (n = 1, 3.0%) and WebQ (n = 1, 3.0%). Two studies did not specify which online program was used.

### Additional methods of data collection

Studies commonly used methods in addition to Q-sorting; more than half (n = 165, 57.1%) used a post-sorting interview, asked additional questions, or allowed for comments to be made on how participants sorted the statements. These post-sorting methods involved enquiring about participants’ placements of cards, particularly at the extreme rankings. Twenty-three studies (8.0%) involved surveys and questionnaires, 14 studies conducted interviews on topics related to the focus of the Q-study but not explicitly about the card placement (4.8%), and 13 studies involved focus groups or group discussions (4.5%). Other methods or analyses included observations, additional statistical analysis, think-aloud tasks, and validity testing.

Two studies [31, 118] (0.7%) translated their Q-methodology findings into a survey, referring to the process as ‘Q2S’. Through a Q-methodological study, Baker et al. [31] identified three viewpoints (factors) on healthcare priorities and resource allocation. In the same article, they then adapted the Q-sorting task into a survey to identify the distribution of each of the three viewpoints in a larger population. Leggat et al. [118] identified two factors regarding the content of clinical supervision for allied health staff. The results were used to develop a survey on the content, outcomes and understanding of clinical supervision.

### Analysis

Over half of the studies (n = 164, 56.7%) provided complete details of the analysis, reporting on the type of factor extraction, rotation and analysis program used. Of the included studies, 116 (40.1%) partially reported the details of their analysis and nine studies (3.1%) did not specify details about the analysis. Two of the included studies [44, 119] (0.7%) carried out second order factor analysis to identify ‘super factors’^1^ or higher-order factors. Wong, Eiser [119] identified three moderately correlated super factors that influenced physician end-of-life care decisions— “patient-focused beneficence”, “patient- and surrogate-focused”, and “best interest guided by ethical principles”—from 17 first order factors. Dennis et al. [44] compared real and ideal hospital environments, identifying three super factors—“Professional”, “Personal” and “Constrained”—from three real and three ideal first order factors.

#### Type of factor extraction

Principal Component Analysis (PCA) was used in 110 studies (38.1%) and centroid analysis was used in 91 studies (31.5%). An additional study [120] (0.3%) first used centroid factor analysis in the assessment of General Practitioners’ perceptions of irritable bowel syndrome. As this indicated the presence of one dominant factor, the authors re-analysed the data using PCA. In 87 of the studies (30.1%), the type of factor extraction used was not reported or was unclear.

#### Rotation

Varimax rotation was used in 206 studies (71.3%) and by hand/manual rotation was used in four (1.4%). Two additional studies [121, 122] (0.7%) used both varimax and manual rotation and two others [123, 124] (0.7%) used oblimin rotation. In their study of medical students’ attitudes toward kidney physiology and nephrology, Roberts et al. [122] explained that they initially used varimax rotation to simplify the factor solution and then manually rotated it in order to better separate students with different attitudes. Skorepen et al. [121] did not provide justification for undertaking both manual and varimax rotation in their study of suffering and dignity in psychiatric hospitals. In two studies [39, 86] (1.0%), the unrotated solution was accepted. Eaton [86] explained that in their study of good birthing experiences an unrotated factor solution was accepted as a dominant factor was revealed by the factor analysis, accounting for a high amount of the study variance. In Paige and Morin’s study [39] on nurse educators’ perspectives of simulation design, the factor solution was initially rotated, however, as this resulted in highly correlated factors, the unrotated factor solution was accepted. Seventy-three studies (25.3%) did not clearly report the type of rotation used, if any.

#### Analysis program

The most commonly used analysis program was PQMethod (n = 186, 64.4%), followed by PCQUANL/QUANL (n = 36, 12.5%) and PCQ (n = 21, 7.3%). Other programs included SPSS (n = 9, 3.1%), Q-Assessor (n = 5, 1.7%), QMethod (n = 4, 1.4%), Qanalyze (n = 1, 0.3%), SAS (n = 1, 0.3%) and STATA software (n = 1, 0.3%). One study [125] (0.3%) used both SPSS and Q-com to analyse data. In 24 of the studies (8.3%), the program used to analyse data was not reported.

### Q-study implications

Authors’ spoke about the implications of their Q-study findings in terms of informing and influencing working and teaching practices, education curricula, clinical treatments, care delivery, policy and protocols, and staff members’ and patients’ wellbeing, while taking into consideration underlying cultural, social, religious, contextual and ethical factors. To more specifically demonstrate how Q-methodology has been used in studies of healthcare and what implications these studies have for clinical practice and/or policy, we present three examples of included studies (Table 3).

**Table 3:** Examples of included studies

**Publication**	Clarke & Holt^46^	Killam, Mossey, Montgomery & Timmermans^109^	Protiére, Baker, Genre, Goncalves & Viens^110^
**Study aim**	To identify and explore the perspectives of nurses and other multidisciplinary stroke team members on nurses’ practice in stroke rehabilitation	To explore undergraduate baccalaureate nursing students’ understanding of clinical safety	To elicit stakeholders’ viewpoints about the dimensions at stake in determining marketing authorisation (MA) and about the processes used to grant MA (including whether the cost of the treatment should be considered in the MA procedure)
**Topic**	Perceptions of treatment/change/intervention	Education	Cancer
**Development of the Q-set**	Data from previous study	Refined from a concourse used in a previous Q study	Review of the literature; semi-structured interviews
**Number of statements in Q-set**	32	43	34
**Ranking scale and anchors**	Most disagree (-4) to Most agree (+ 4)	Most disagree (-5) to Most agree (+ 5)	Most disagree (-4) to Most agree (+ 4)
**Example statement**	Nurses are the most appropriate professional to liaise between stroke survivors, families and the stroke unit team	The student makes independent clinical decisions beyond his/her competency	If a treatment can prolong lifespan, even by one month, it should be given MA whatever its cost to society
**Delivery method**	Face-to-face	Face-to-face	Online (Flash Q)
**Participants**	63 healthcare employees regularly working with/visiting patients on a stroke unit (registered nurse, healthcare assistant, therapist, physician, dietician, social worker, clinical psychologist, orthoptist)	68 first year nursing students	48 healthcare employees (oncologists, healthcare decision makers, individuals from the pharmaceutical industry) and 104 consumers (patients; members of the general population)
**Analysis**	PCA; Varimax; PQMethod	Centroid; Varimax; PQMethod	PCA; Manual rotation; PQMethod
**Other methods**	Post-sorting questions/additional comments; semi-structured interviews	Post-sorting group discussion	Post-sorting questions/additional comments
**Factors/viewpoints**	n = 4	n = 4	n = 3
	1. Integrate rehabilitation principles in routine nursing practice; 2. Physical care activity takes priority over rehabilitation principles; 3. Support the wider stroke team to provide stroke rehabilitation; 4. Be cautious about nurse’ engagement in stroke rehabilitation practice	1. Overwhelming sense of inner discomfort; 2. Practicing contrary to conventions; 3. Lacking in professional integrity; 4. Disharmonising relations	1. Quality of life, opportunity cost and participative democracy; 2. Quality of life and patient centeredness; 3. Length of life
**Variance explained**	66%	Not reported	44%
**Study implications**	Results gave insight into similarities and differences in viewpoints amongst clinical staff on nursing practices in stroke units. Study findings demonstrated the need for structured competency-based multidisciplinary training in rehabilitation skills to facilitate partnerships between registered nurses and healthcare assistants in stroke rehabilitation	The study found that compromised clinical safety is a complex concept involving personal, professional and programic variables. The authors suggested that study findings could be used to develop learning environments that are safety-oriented and student-centred	Based on the study findings, the authors indicated that there is a need for transparency and re-evaluation of treatments after they have received marketing authorisation. They also suggest that authorisation criteria should include a greater focus on quality of life in the context of advances cancer care

#### Factor solutions

The numbers of factors identified by studies ranged from 0 (i.e., no identifiable factors) to 21 factors, which takes into account the total number of factors across participant groups. For example, Protière et al. [101] identified four factors for patients and four factors for healthcare professionals in their study of marketing authorisation procedures for advanced cancer drugs, and therefore, the total number of factors recorded was eight. The most common number of factors identified by included studies was four (n = 100, 34.6%).

Some studies [98, 101, 106, 109, 111, 126, 127] reported multiple study variances (e.g., for different participant groups). In this case, each reported variance was considered separately, and they ranged from 20.0% to 90.8% (M = 53.4%, SD = 11.6). Ninety studies (31.1%) did not report the total variance explained.

## Discussion

This review explored how Q-methodology has been used in healthcare research. Through a comprehensive search and rigorous screening process, 289 studies were identified that used Q-methodology to study topics in healthcare. We captured data on how these studies were conducted, as well as information on the value and applications of Q-methodology to healthcare research.

### Use of Q-methodology in healthcare research

In terms of the materials used to conduct Q-methodological studies in healthcare, we found several strategies for developing Q-sets, with literature reviews being most common. Although there was some variation in ranking scale ranges, the majority of studies utilised anchoring based on agreement, indicating most Q-studies in healthcare collect information on how people understand a topic (e.g., compulsory mental healthcare [85] or infant immunisation [128]), rather than prioritisation, as priority and importance anchors had much lower usage.

Face-to-face administration of the Q-sorting task was by far the most common approach to data collection. In many instances, this occurred in conjunction with another method like interviews/post-sorting questions, [129, 130] focus groups/group discussions, [131, 132] a think-aloud task, [23] or observations, [88, 133] to capture information that clarified participants’ placement of the Q-set or provide additional insights into the topic. Perhaps due to the sensitive nature of many aspects of healthcare, mail-out and online forms of collecting Q-data were less common, although several software programs were used for online data collection. In terms of analysis, both PCA and centroid techniques were well represented approaches to factor extraction, while varimax rotation was used in the overwhelming majority of studies for rotation, suggesting a preponderance of exploratory rather than theoretically driven Q-studies in healthcare.

### Value of Q-methodology in healthcare research

Healthcare is a complex system, [1, 134, 135] featuring ‘wicked problems’ (e.g., sustainability, patient safety, chronic disease management, and health inequity) that have multiple interacting issues and are socially constructed from the standpoint of different observers [136, 137]. Q-methodology can be used to explore such issues because Q-sorting invites individuals to model their subjectivity by considering multiple issues in relation to each other. Our results highlight that the method is also highly suited for use with different healthcare stakeholders. Q-studies ranged from exploring the perspectives of one stakeholder group (e.g., Intensive care unit nurses [138]) to many (e.g., nurses, alternative therapists, general practitioners, surgeons, allied health professionals, mental health professionals and patients [87]), with approximately one tenth of studies examining consumer perspectives simultaneously with providers. Even where a seemingly homogenous group of participants was involved, multiple perspectives (factors) on the topic were typically uncovered.

Included studies used Q-methodology to tackle a diverse range of healthcare topics, although almost half were broadly in the areas of health professional education, mental healthcare, or nursing practice. Other common topic areas were those that have clear emotional and moral dimensions, including chronic disease, end-of-life care, resource allocation, and gender, sexual and reproductive health. As we and others have noted, [42, 55] these are the kinds of matter considered highly suited to investigation using Q-methodology. In this vein, many authors of articles we reviewed cited the value of using Q-methodology in being able to study complex and contentious topics in which there were likely to be differing opinions [51–54]. Other reasons given for using Q-methodology that are particularly pertinent to healthcare research included the way it allowed minority populations, or participants who often do not have a voice, or find it difficult to participate in other forms of research, to contribute their perspective.

### Implications

Studies using Q-methodology in healthcare research have increased over the past 5 years, suggesting a growing interest and acceptance of what was once considered a niche psychological tool [14]. However, the use of Q-methodology in healthcare remains limited when compared to more traditional approaches such as questionnaire surveys. Undoubtedly some healthcare topics are better suited to surveys, but where exploration of a contested or complex issue is required, and holistic understanding of the different perspectives that converge on that topic are desired, Q-methodology should be considered. Moreover, depending on the research questions, Q-methodology may work in tandem with surveys. We reviewed two studies using the emergent design of Q2S, where different perspectives are first identified, using Q-methodology, and then the extent of their distribution in the population is investigated through surveys.

Our review found that Q-methodological research was spread across a large of number of journals. To some extent this reflects the diverse topics that Q has been used to study, however, with very few journals publishing more than one or two studies, it is likely the method remains novel and unfamiliar to many healthcare researchers. The greatest take-up appeared to be in nursing, including nursing education, which is perhaps not surprising given the longstanding and widespread acceptance of interpretive methods in nursing research [139]. A large proportion of Q-methodological research was conducted in the UK, the home of Q, followed by The US, although researchers in countries where English is not the main language have also frequently used the approach, such as South Korea.

For the most part, the studies we reviewed provided information necessary to understand how Q had been applied and the significance of the results. However, there were many occasions of authors not providing all details such as how they derived their Q-set, what types of analyses they performed, and the amount of variance explained. To facilitate increasing use of Q, and publication of Q-methodological studies, we offer a checklist of details that should be included when reporting these studies (Table 4).

**Table 4:** Checklist of information to include when reporting a Q-methodological study

How items/statements for the Q-set were collected
How the statements were refined and reduced to produce the draft and final Q-set
The number of statements in the final Q-set
What, if any, piloting was done and what the results were
The materials used for the Q-sorting task including the ranking scale and anchors
How the Q-sorting task was administered
What, if any, other methods were used in conjunction with Q-sorting, and how the data captured by these methods was used in relation to Q-data
The techniques used for factor extraction and rotation
The software programs used to administer and/or analyse the data
The information used to decide the number of factors to extract, rotate and interpret
The amount of variance explained by the factor solution
The processes for interpreting the factors
A rich narrative for each factor that explains the shared meaning it represents, supported by Q-set statements, and participant quotes where available

### Strengths and limitations

This review had a comprehensive strategy involving searches of three large academic databases, using a range of keywords associated with both Q-methodology and healthcare. A rigorous approach to reviewing was established though regular discussions between reviewers. Data verification was conducted by one reviewer (KL), who standardised data entry and clarified any inconsistencies by consulting included studies. It is possible that some studies that have used Q-methodology in healthcare were not captured, such as those that used less specific terms (e.g., narratives, viewpoints, or factor analysis) in the title and abstract. This has not limited our capacity to identify a number of trends among included studies. Following scoping review conventions, a formal quality assessment of included articles was not undertaken.

## Conclusions

Q-methodology is an approach to studying complex issues that to some extent simplifies complexity by reducing participants’ viewpoints to a smaller number of common perspectives through inverted factor analysis. Nevertheless, the method’s holistic approach to data collection, analysis and interpretation retains the nuances of different perspectives. Arguably, this makes Q-methodology an ideal method for studying the kinds of complex, divisive and ethically fraught issues that are commonplace in healthcare. Our review demonstrated increasing use of the approach in healthcare, however, this research is diffuse, spread across a large number of journals and topic areas, suggesting Q-methodology is still fairly novel. We have highlighted commonalities in how the method has been used, areas of application, and the potential value of the approach, which may lead to increased use of Q-methodology in the future.

## Supplementary Information

**Additional file 1.** PRISMA-ScR checklist.

**Additional file 2.** Keyword search in Web of Science Medline.

**Additional file 3.** Full list of studies included in the review and details of data items charted.

## Data Availability

The datasets used and/or analysed during the current study are available from the corresponding author on reasonable request.

## Abbreviations

PRISMA: Preferred Reporting Items for Systematic Review and Meta-analyses
Q2S: Q-methodology to survey
UK: United Kingdom
US: United States

## Footnotes

1 Super factors are the resulting factors from a second order factor analysis, in which the first order factor arrays (ideal Q-sorts computed for each factor) are considered Q sorts.
