# Supplementary material for: A scoping review of Q-methodology in healthcare research
Source: BMC Med Res Methodol. 2021 Jun 21;21:125. doi: 10.1186/s12874-021-01309-7 (PMC8215808; doi:10.1186/s12874-021-01309-7)
Supplement: Supplementary file 2 — Additional file 2. Keyword search in Web of Science Medline. [file 12874_2021_1309_MOESM2_ESM.docx]

***Web of Science Search***

| **Search Line** | **Search details** | **No. citations** |
| --- | --- | --- |
|  | TS=("health care" OR healthcare OR "health-care" OR medic* OR nurs* OR "health services" OR patient OR hospital* OR clinic* OR "acute care" OR "primary health" OR "primary care" OR "general practice")  Indexes=MEDLINE Timespan=1950-2019 | 10,979,407 |
| 1. 2. | TS=("q methodology" OR "q-methodology" OR "q method" OR "q-method" OR "q sort" OR "q-sort")  Indexes=MEDLINE Timespan=1950-2019 | 38,694 |
| 1. 3. | #1 AND #2 | 637 |
